# The mechanism and pharmacodynamics of 2-((1H-indol-3-yl)thio/sulfinyl)-N-pheny acetamide derivative as a novel inhibitor against human respiratory syncytial virus

**DOI:** 10.1080/14756366.2022.2123804

**Published:** 2022-09-21

**Authors:** Ningning Cheng, Nan Jiang, Yuanhui Fu, Zhuxin Xu, Xianglei Peng, Jiemei Yu, Shan Cen, Yucheng Wang, Guoning Zhang, Yanpeng Zheng, Jinsheng He

**Affiliations:** a College of Life Sciences and Bioengineering, Beijing Jiaotong University, Beijing, China; b Institute of Medicinal Biotechnology, Chinese Academy of Medical Science and Peking Union Medical College, Beijing, China

**Keywords:** 2-((1H-indol-3-yl)thio/ sulfinyl)-N-pheny acetamide derivative, respiratory syncytial virus, antiviral mechanism, pharmacodynamics, *in vivo* imaging

## Abstract

Human respiratory syncytial virus (RSV) is a leading cause of lower respiratory tract infection worldwide. Until now, there are no licenced vaccines or effective antiviral drugs against RSV infections. In our previous work, we found 2-((1H-indol-3-yl)thio/sulfinyl)-N-pheny acetamide derivatives (4-49 C and 1-HB-63) being a novel inhibitor against RSV *in vitro*. Here, we explored the underlying mechanism of 2-((1H-indol-3-yl)thio/sulfinyl)-N-pheny acetamide derivatives to inhibit RSV replication *in vitro* and disclosed that 4–49 C worked as the inhibitor of membrane fusion and 1-HB-63 functioned at the stage of RSV genome replication/transcription. Yet, both of them could not inhibit RSV infection of BALB/c mice by using RSV-Luc, *in vivo* imaging and RT-qPCR analyses, for which it may be due to the fast metabolism *in vivo*. Our work suggests that further structural modification and optimisation of 2-((1H-indol-3-yl) thio/sulfinyl)-N-pheny acetamide derivative are needed to obtain drug candidates with effective anti-RSV activities *in vivo*.

## Graphical Abstract

**Figure:**
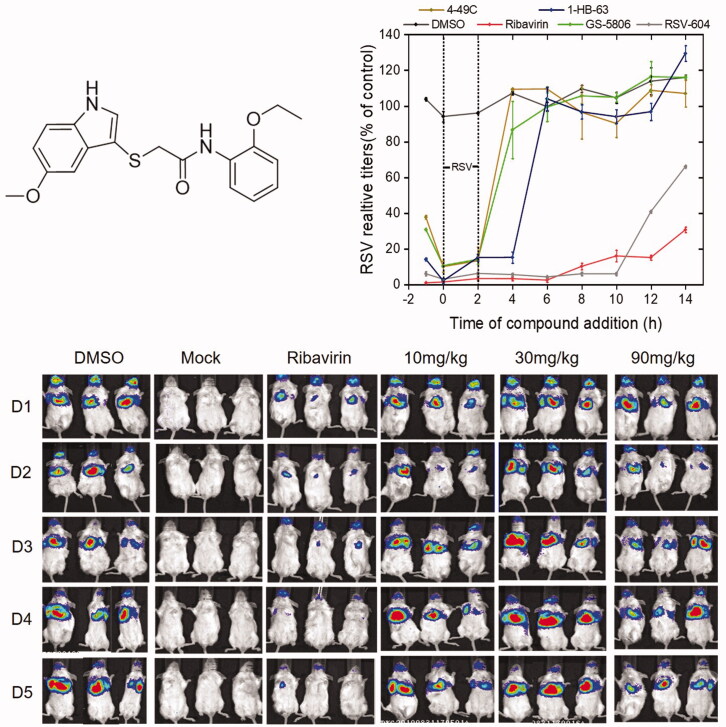

## Introduction

Human respiratory syncytial virus (RSV) is an enveloped, negative-sense, single-stranded RNA virus and belongs to the Pneumovirus family and the Orthopneumovirus genus^1–4^. It is 15.2 kb in length and contains 10 genes encoding 11 proteins, NS1, NS2, N, P, M, SH, G, F, M2-1, M2-2, and L^5–8^. RSV is a leading cause of lower respiratory tract infection in nearly all children by 2-year-old globally and it was estimated that there were 3.2 million hospitalised cases and about 60,000 deaths annually^9–12^. RSV is also an important pathogen of severe lower respiratory tract disease in the elderly and adults with immunodeficiency disorders^13^^,^^14^. Unfortunately, there is no safe and effective vaccine licenced against RSV infection. Only ribavirin and palivizumab are the available drugs for the treatment of RSV infection^15–18^. Yet, being a broad-spectrum antiviral drug, ribavirin is not recommended due to its poor efficacy and side effects. Palivizumab, a costly humanised monoclonal antibody for RSV, can only be used to prevent RSV infection for the young and high-risk children born prematurely, with chronic lung disease or congenital heart disease^19–21^. Therefore, the development of safe and effective anti RSV drugs has important clinical significance^22–24^.

In our previous work, we established an rRSV-mGFP high-throughput screening platform for the potential compounds active against RSV^25^, from which 29 compounds were identified as potent RSV inhibitors and two of them were selected for further structural optimisation following the evaluation of their toxicity, druggability and feasibility of synthesis^26^. Among the developed 35 derivatives, 4–49 C and 1-HB-63, both of the derivatives of 2-((1H-indol-3-yl)thio/sulfinyl)-N-pheny acetamide, exhibited excellent activity against RSV and IAV^26^. In this paper, we investigated the underlying mechanism *in vitro* and pharmacodynamics *in vivo* of the two 2-((1H-indol-3-yl)thio/sulfinyl)-N-pheny acetamide derivatives as inhibitor against RSV.

## Materials and methods

### Cells and viruses

HEp-2 cell and wild-type subgroup A RSV Long strain (wtRSV) were purchased from ATCC (Rockefeller, MD, USA). BHK-T7 was a gift from Prof. W. Y. Zhu (National institute for viral disease control and prevention, Chinese centre for disease control and prevention, Beijing, China). And RSV-Luc was kindly provided by Dr. Marie-Anne Rameix-Welti (University of Versailles Saint-Quentin, Versailles, France). RSV-Luc and wtRSV were propagated in HEp-2 cells using DMEM (Gibco BRL, Gaithersburg, MD, USA) with 2% FBS (Hyclone, Logan, UT, USA), L-glutamine (2 mM final concentration), penicillin G (40 U/ml), streptomycin (100 μg/ml) and 0.2% sodium bicarbonate. Then RSV-Luc and wtRSV were purified by sucrose ultracentrifugation and titrated for infectivity by immunoplaque assay, and expressed as plaque-forming units (PFU) per ml.

### Drugs and compounds

P13 and Ribavirin were purchased from Topscience Company (Shanghai, China). GS-5806 (fusion inhibitor) and RSV-604 (N protein inhibitor) were purchased from MCE (MedChemExpress, Monmouth Junction, NJ, USA). D-luciferin potassium salt was purchased from Sigma (Sigma, St. Louis, MO, USA). Ribavirin was dissolved in deionised water, and the synthetic compounds (4–49 C and 1-HB-63) were dissolved in DMSO to prepare the working solution.

### Time-of-addition assay

The time-of-addition assay was performed as follows according to our previous work and literature^25^^,^^27^. HEp-2 cells were plated in 96 well plates at 1.5 × 10^4^ cells per well in 100 µL culture medium and incubated for 24 h (37 °C, 5% CO_2_). Then, HEp-2 cells were infected with wtRSV at a multiplication of infection (MOI) of 5 for 2 h (37 °C, 5% CO2). The cells were washed with PBS and the media containing drugs and compounds of 4–49 C, 1-HB-63, RSV-604, GS-5806 and Ribavirin at 1 mM, 200 μM, 10 μM, 1 mM and 100 μM, respectively, were added at −1, 0, 2, 4, 6, 8, 10, 12 and 14 h post infection. After the treated cells were further cultured and collected at 24 h post infection, the total RNA was isolated from the HEp-2 cells using TRIzol reagent (Thermo Fisher Scientific, Waltham, MA, USA) and reverse transcribed with GoScript Reverse Transcriptase kit (Promega, Madison, WI, USA). Then, RT-qPCR was performed as described previously^28^.

### RSV minigenome assay

BHK-T7 cells were seeded into 24 well plates at a density of 2 × 10^4^ cells/well and incubated for 24 h. Then the RSV minigenome plasmid encoding firefly luciferase (pBR322B-RSV-Gluc) was mixed with the helper plasmids (pCITE-N, pCITE-P, pCITE-L and pCITE-M2-1) in an Eppendorf tube containing 250 μL Opti-MEM. Meanwhile, the transfection reagent of Lipofectamine 2000 (Invitrogen, CA, USA) was diluted in another tube containing 250 μL Opti-MEM and incubated 5 min at room temperature. Then, the plasmids were mixed with the transfection reagent and incubated at room temperature for 30 min. After that, the BHK-T7 culture media were replaced with the DNA-lipo-optiMEM mixture and incubated for 4 ∼ 6 h (37 °C, 5% CO_2_). Then the DNA-lipo-optiMEM mixture was replaced with media containing the compounds (4–49 C at 1 mM and 1-HB-63 at 200 μM) and further incubated for 24 h (37 °C, 5% CO_2_). Finally, after the supernatant of the BHK-T7 cells was transferred into 96 well plate, the Gluc activity was determined by using LUMI star (BMG ABTECH, Ortenberg, Germany) following the manufacturer’s instructions after adding the chromogenic substrate of Gaussia Luciferase Flex Assay Kit (NEB, Ipswich, USA). The results were recorded by photography and/or evaluated with OPTIMA software (BMG LABTECH). The resultant signal strength was quantified as relative light unit (RLU).

### In vivo mouse imaging

Female BALB/c mice of 6-8 weeks were purchased from Charles River Laboratories, Beijing, China and randomly divided into different groups with six mice in each group. All animal experiments were performed in compliance with protocols approved by the Institutional Animal Care and Use Committees of Tsinghua University (No. 17-DZJ1). The compounds (DMSO and ribavirin of 90 mg/kg was used as negative and positive control, respectively) with three different dose groups of 10, 30 and 90 mg/kg were administered to mice by intraperitoneal (i.p.) injection or oral route (anaesthesia with 2% halothane) at zero day and 1 h prior to the mice infected with 50 μL RSV-Luc (1 × 10^6^ PFU), and then once a day for 5 consecutive days post RSV-Luc infection. For the *in vivo* mouse imaging, 50 μL PBS containing D-Luciferin Potassium Salt (75 mg/kg) was administered to BALB/c mice via nasal drip at days 1, 2, 3, 4 and 5 since RSV-Luc infection, and 5 min later, the whole-body images of BALB/c mice were acquired using an IVIS Lumina II *in vivo* imaging system (PE, Waltham, MA, USA). And the signal intensity was quantified as the total flux (photons/seconds) using Living Image software (PE).

### Determination of RSV titre from lung tissues

Following the experiment of the *in vivo* mouse imaging mentioned above, the mice were sacrificed. The lung tissues were collected, weighed, quickly frozen under liquid nitrogen and ground with a tissue homogeniser. Then the total RNA was extracted by Trizol from 100 µL homogenates. The viral RNA sample (1 µL) was mixed with the SYBR Green supermix (10 µL), plasmid pMD-18T-N (1 µL, copy number from 1 × 10^7^ copies/μL to 1 × 10^5^ copies/μL), the upstream and downstream primers, respectively (10 µM, 1 µL) and water (1 µL). Reactions were performed in SLAN-96P Real-Time PCR System (Shanghai Hongshi Medical Technology) as follows: 42 °C for 60 min (for reverse transcription), 95 °C for 1.5 min, 95 °C for 15 s and 60 °C for 1 min, for 40 cycles. The reaction data were analysed by determining the threshold cycle (Ct).

### Pharmacokinetic study in rat

Six male Sprague − Dawley (SD) rats, randomly divided into two groups of 3 each, were treated with 4–49 C predissolved in 5% DMSO, 5% Solutol and 90% Saline by intravenous injection (iv, 5 mg/kg) and in 5% DMSO and 95% (0.5%MC) by intragastric administration (ig, 10 mg/kg), respectively. Blood samples (100 μL) were collected into K2-EDTA tubes by retro‐orbital bleeding under anaesthesia using isoflurane at 0.033, 0.083, 0.25, 0.5, 1, 2, 4, 7 and 24 h after dosing. And then plasma samples were obtained by centrifuging at 5000 rpm for 10 min at 4 °C and stored at −80 °C until analysis using HPLC–MS/MS.

### Data processing and statistics

Graphpad prism 7 software was used for statistical analysis. The t-test was used when there was one comparison, while one-way ANOVA was performed for multiple comparisons. *P* < 0.05 was considered statistically significant.

## Results and discussion

**Figure 1.**
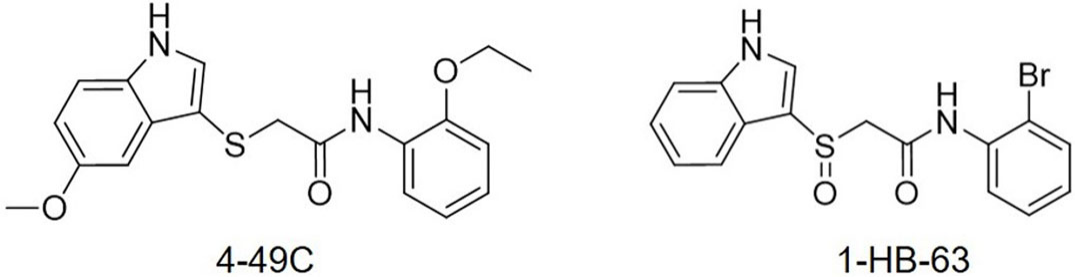
Structure of 2-((1H-indol-3-yl) thio/sulfinyl)-N-pheny acetamide derivative.

**Figure 2.**
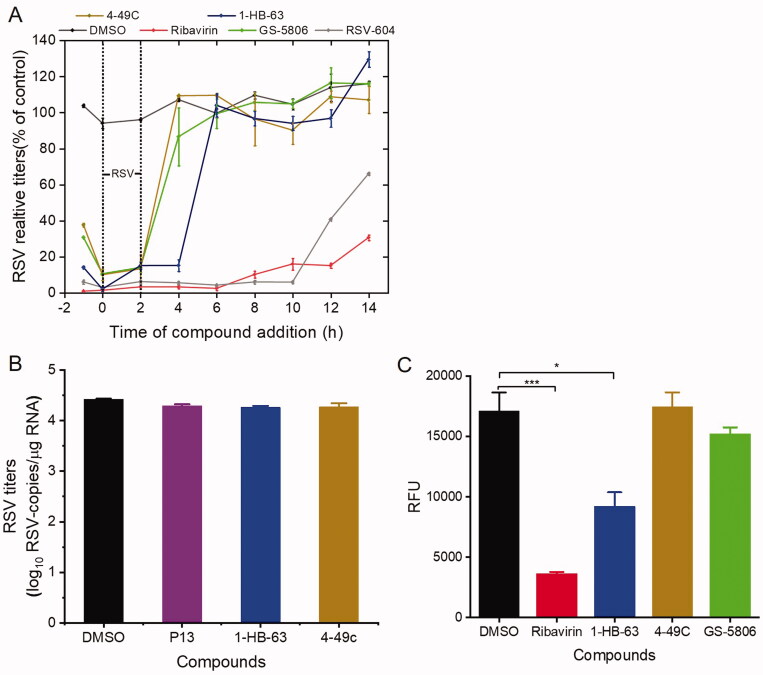
Investigation on the underlying mechanism 4–49 C and 1-HB-63 inhibiting wtRSV replication. (A) Time-of-addition analyses of 4-49C and 1-HB-63 replication. The HEp-2 cells were infected with wtRSV at a multiplication of infection (MOI) of 5 for 2 h (37 °C, 5% CO2). The cells were washed with PBS and the media containing drug (4-49C, 1-HB-63, RSV-604, GS-5806 and Ribavirin at 1 mM, 200 μM, 10 μM, 1 mM and 100 μM, respectively.) were added at −1, 0, 2, 4, 6, 8, 10, 12 and 14 h separately and incubated till 24 h post infection. Then, the cells were harvested and lysed for RNA extraction and RT-qPCR assay. The results were a representative of three independent experiments, and shown as means ± standard and expressed as % negative control, although in many cases the bars for the standard deviation were obscured by the symbols given the small margin of values. (B) RSV adsorption analyses. After HEp-2 cells seeded into 24 well plates for 24 h, the cold media containing RSV (5 MOI) and compounds of 4–49C, 1-HB-63 and P13 at 1 mM, 200 μM and 100 μM, respectively, was added and incubated for 1 h at 4 °C. Then, the total RNA was isolated and RT-qPCR was performed. (C) RSV minigenome assay. After 24 h, we cotransfected RSV minigenome plasmid of pBR322B-RSV-Gluc with four auxiliary plasmids into BHK/T7 cells and detected the expression of Gluc in the supernatant. The results were shown as means ± standard deviations for three independent experiments, *p < 0.05, ***p < 0.001.

The time-of-addition assay, hence, was carried out to specify replication stage (s) at which 4–49 C and 1-HB-63 (Figure 1) inhibit RSV infection, respectively. As the results shown in Figure 2(A), compared with DMSO (negative control), ribavirin (genome replication stage-targeted inhibitor), RSV604 (N protein inhibitor), and GS-5806 (fusion inhibitor), 4-49 C significantly inhibited virus replication when added at the time points of −1, 0 and 2 h, similarly to the GS-5806 having inhibition activity only when added up to 0 and 2 h. While 1-HB-63 significantly inhibited virus replication when added at the time points of −1, 0, 2 and 4 h. Thus, the results indicated that 4-49 C inhibits virus replication in the initial stage of RSV infection or before entry of the targeted cells, and 1-HB-63 acts in the late stage of RSV infection or after entering the cells.

Further, the experiment on whether the chemicals inhibited RSV replication by affecting the attachment of RSV to cells was performed (Figure 2(B)). We found that neither of 4-49 C and 1-HB-63 inhibited RSV replication by preventing virus adsorption. Then, RSV minigenome assay by co-transfecting RSV minigenome plasmid of pBR322B-RSV-Gluc and four auxiliary plasmids into BHK/T7 cells was also carried out. After 24 h, the expressed Gluc in the supernatant of BHK/T7 cells was detected and shown in Figure 2(C). Compared with the negative control of DMSO and the positive controls of GS-5806 and ribavirin, the expression of Gluc gene could not be inhibited by 4–49 C, similar to GS-5086, which is sharply contrast to the effect of 1-HB-63 or ribavirin, thus suggesting that 4–49 C and 1-HB-63 may play anti-RSV roles by inhibiting membrane fusion and by inhibiting RSV replication at the stage of RSV genome replication/transcription, respectively. Therefore, the study described above is invaluable to clarify the underlying mechanism of these two anti-RSV compounds, 4–49 C and 1-HB-63, and lays an important foundation for further investigation of their *in vivo* antiviral activities.

Although BALB/c mouse is semi-permissive to RSV infection, the weight loss and the lung pathological changes as well as the virus replication in lung tissue are observable following the infection intranasally. In addition to the traditional immuno-plaque or RT-qPCR assays, *in vivo* non-invasively mouse imaging can be used to investigate the pharmacokinetics and pharmacodynamics of anti-RSV drugs. Thus, in this paper, RSV-Luc, the recombinant RSV expressing the luciferase reporter gene, was selected to infect BALB/c mice and combined with an *in vivo* imaging technology to monitor the *in vivo* pharmacodynamics of 4–49 C and 1-HB-63.

**Figure 3.**
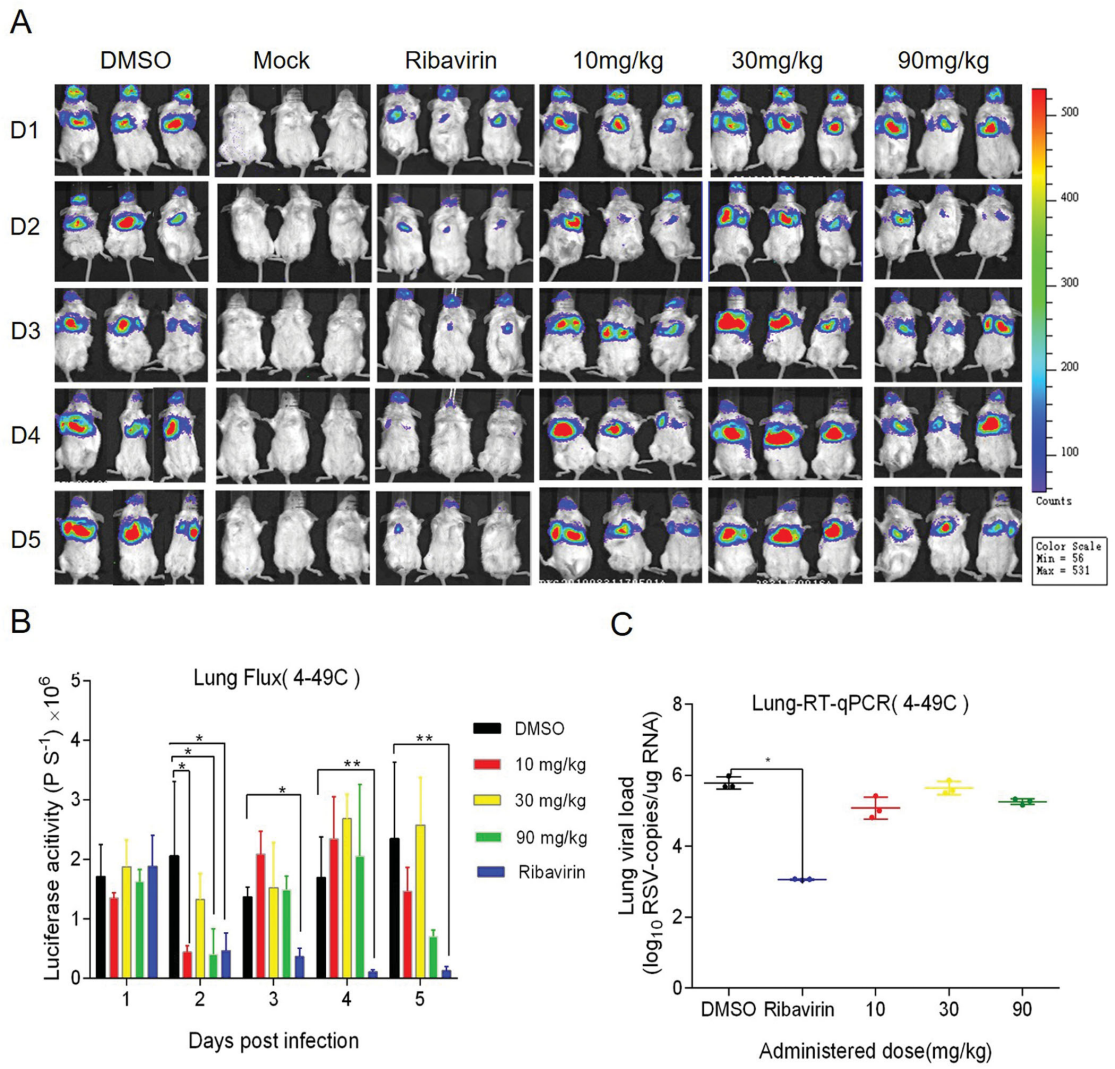
The *in vivo* anti-RSV activity of 4–49 C administered by intraperitoneal injection. A, *in vivo* imaging of BALB/c mice following infection of RSV-Luc and treatment with 4-49C pre- and post-infection; B, the replication levels of RSV-Luc, represented by luciferase activity, in each group of mice following infection and treatments at different dosages of 10, 30 and 90 mg/kg and at different time points; C, viral titres in the lung tissues from each group of mice analysed by RT-qPCR. The results were shown as means ± standard deviations, *p < 0.05, **p < 0.01.

**Figure 4.**
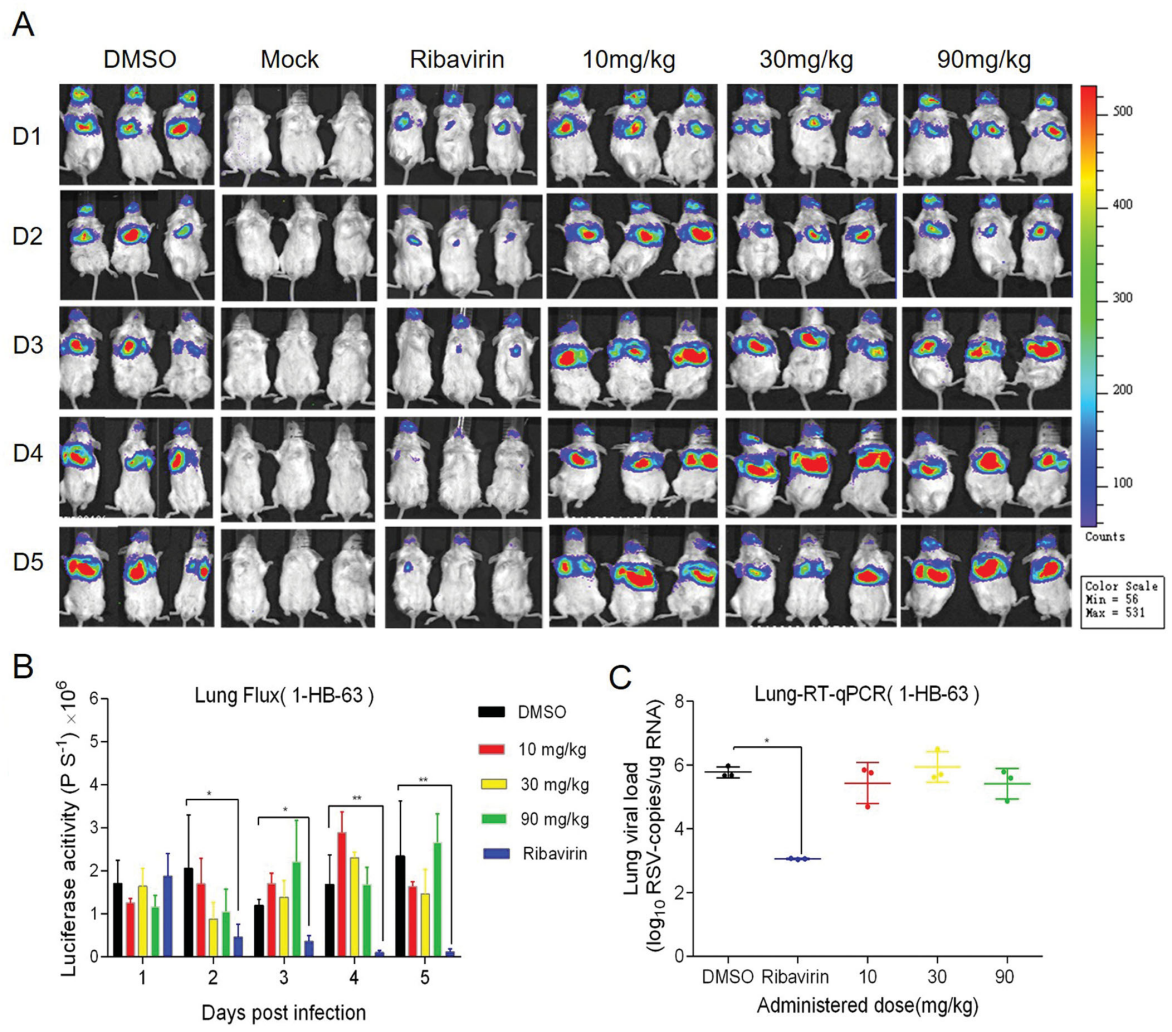
The *in vivo* anti-RSV activity of 1-HB-63 administered by intraperitoneal injection. A, *in vivo* imaging of BALB/c mice following infection of RSV-Luc and treatment with 1-HB-63 pre- and post-infection; B, the replication levels of RSV-Luc, represented by luciferase activity, in each group of mice following infection and treatments at different dosages of 10, 30 and 90 mg/kg and at different time points; C, viral titres in the lung from each group of mice analysed by RT-qPCR. The results were shown as means ± standard deviations, *p < 0.05, **p < 0.01.

Female BALB/c mice of 6–8 weeks were randomly divided into different groups with 6 mice in each group. All animal experiments were performed in compliance with protocols approved by the Institutional Animal Care and Use Committees of Tsinghua University (No. 17-DZJ1). At day 0, three different doses of 4–49 C and 1-HB-63 (10 mg/kg, 30 mg/kg, 90 mg/kg) were administered to mice by intraperitoneal (i.p.) injection, respectively, with DMSO and ribavirin of 90 mg/kg separately used as negative and positive control. One-hour later, the mice were infected with 50 µL RSV-Luc (1 × 10^6^ PFU). Then two different compounds were administered once a day for 5 consecutive days and the replication levels of RSV-Luc in the lungs of BALB/c mice were observed using an IVIS Lumina II *in vivo* imaging system. As shown in Figures 3(A) and 4(A), except for the groups administered 4–49 C of 10 mg/kg and 90 mg/kg on the second day, the fluorescence signals from the groups both 4–49 C and 1-HB-63 did not display significant differences at any doses and any time points compared with DMSO-treated group, and the results of statistical analysis shown in Figures 3(B) and 4(B) were also proved this. Furthermore, the virus titres in lung tissues at day 5 post-infection were also detected by RT-qPCR and no significant differences were found either between the groups of treatments and DMSO (Figures 3(C) and 4(C)). Thus, the results indicated that 4–49 C and 1-HB-63 injected intraperitoneally had no inhibitory effect on RSV replication in the infected mice.

**Figure 5.**
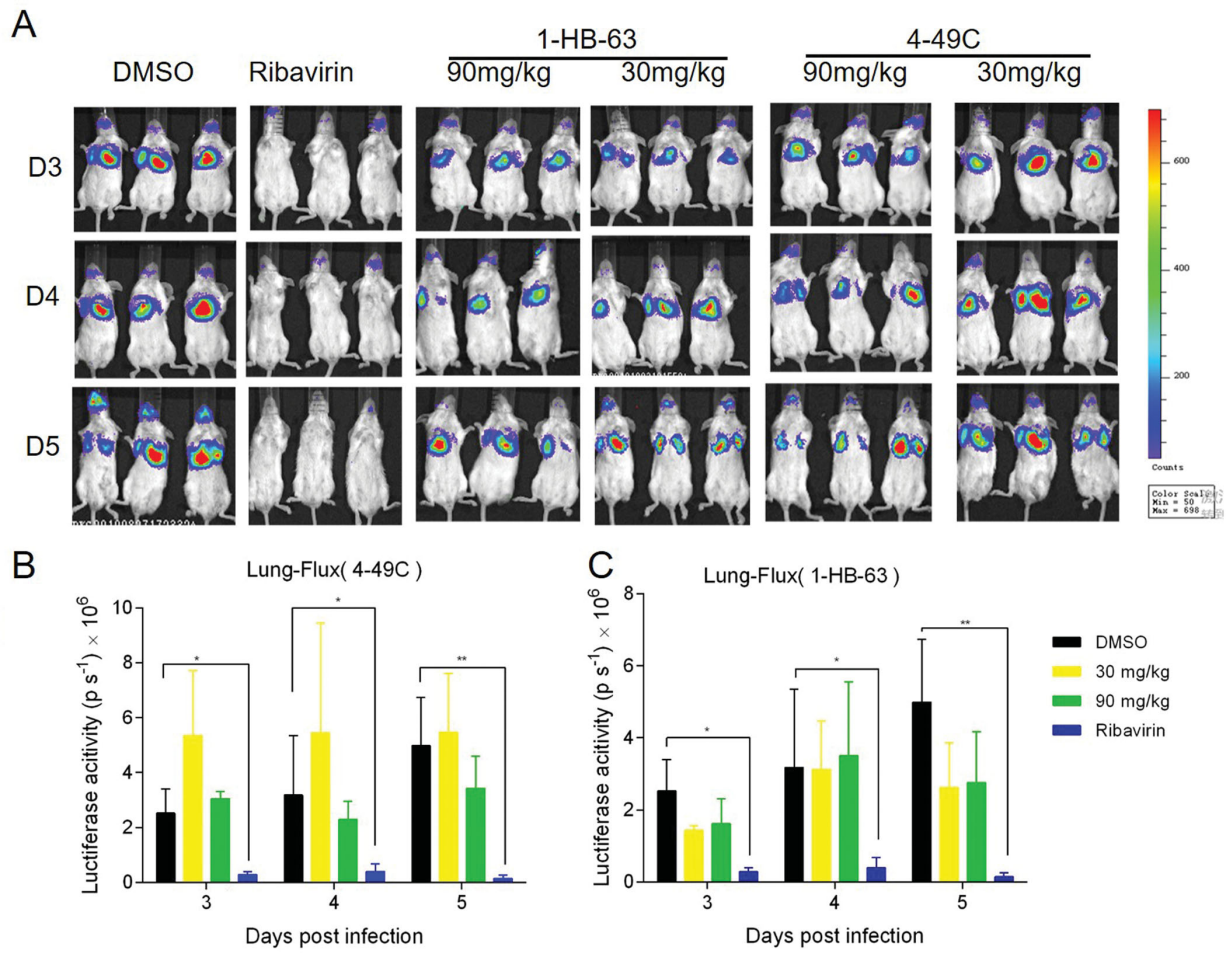
The *in vivo* anti-RSV activity of 4-49 C and 1-HB-63 administered by oral route. A, *in vivo* imaging of BALB/c mice following infection of RSV-Luc and treatment with 4-49 C and 1-HB-63 pre- and post-infection, respectively; the replication levels of RSV-Luc, represented by luciferase activity, in each group of mice following infection and treatments with 4-49C (B) or 1-HB-63 (C) at different dosages of 30 and 90 mg/kg and at different time points. The results were shown as means ± standard deviations, *p < 0.05, **p < 0.01.

In order to further make clear whether the anti-RSV activity of 4–49 C and 1-HB-63 is dependent on the administration route or not, these two compounds were given intragastrically (Figure 5(A)). Except for the doses of 30 mg/kg, 90 mg/kg and gavage twice a day, other procedures were the same as those in intraperitoneal injection experiment. As shown in Figure 5(A–C), compared with DMSO negative control group, the fluorescence signals from the groups of 4–49 C and 1-HB-63 did not exhibit significant differences, which was consistent with the above results by using intraperitoneal injection. Therefore, 4–49 C and 1-HB-63 could not inhibit RSV replication *in vivo* following intragastric application.

To investigate the pharmacokinetic parameters *in vivo*, 4–49 C was selected for the pharmacokinetic experiments in SD rat model. The SD rats were divided into two groups (*n* = 3) and treated by iv at 5 mg/kg dose and by ig at 10 mg/kg dose, respectively. Plasma samples were collected up to 24 h postdosing and the concentration of 4–49 C was measured by using HPLC-MS/MS. As shown in Table 1, the *T*_1/2_ was 0.167 ± 0.3 h and AUC_0-t_ was 69.3 ± 17 h⋅ng/mL after iv administration implying that 4–49 C was rapidly metabolised in plasma. Unfortunately, there is no 4–49 C detected in plasma samples after ig administration (data not shown). The PK results indicate that the poor *in vivo* efficacy of 4–49 C may due to their overly fast metabolism.

**Table 1.** Pharmacokinetic parameters of 4–49 C (iv, 5 mg/kg)

Parameters	Mean ± SD
*K*_el_ (h^-1^)	4.28 ± 0.97
*T*_1/2_ (h)	0.167 ± 0.3
*C*_max_ (ng/mL)	454 ± 110.65
AUC_0-t_ (h·ng/mL)	69.3 ± 17
AUC_0-inf_ (h·ng/mL)	70 ± 17
MRT_IV_ (h)	0.131 ± 0

In summary, 2-((1H-indol-3-yl) thio/sulfinyl)-N-pheny acetamide derivatives as a novel inhibitor against RSV replication were screened successfully, and the underlying antiviral mechanism was elucidated *in vitro*. However, no *in vivo* inhibitive activity on RSV infection was observed based on BALB/c infection model. And the PK results showed that there was a fast metabolism *in vivo* after dosing. Our work proves the difficulty and complexity of drug discovery and development and suggests that further structural modification of 2-((1H-indol-3-yl) thio/sulfinyl)-N-pheny acetamide derivative is needed to improve their stability *in vivo*, and intranasal administration strategy should also be explored.
